# Supplementary material for: Patterns of Intron Gain and Loss in Fungi
Source: PLoS Biol. 2004 Nov 30;2(12):e422. doi: 10.1371/journal.pbio.0020422 (PMC532390; doi:10.1371/journal.pbio.0020422)
Supplement: Table S1 — Also available at http://genes.mit.edu/NielsenEtAl/. (4.3 MB ZIP). [file pbio.0020422.st001.zip › NielsenEtAl/html/1317.html]

AN7567.1.NCU09803.1.MG06356.1.FG01317.1


```
 CLUSTAL W (1.82) Multiple Sequence Alignments - Introns Inserted


Sequence 1: NCU09803.1	266 aa
Sequence 2: MG06356.1	294 aa
Sequence 3: FG01317.1	253 aa
Sequence 4: AN7567.1	275 aa
Alignment Length: 320 aa
Number Identitical Residues: 127 aa
Alignment Score (without introns) 6117


MG06356.1 	MTPPQSIATPAPANEGHAIVLRISTPPIIGSNAFASWPSS2DITTLEEWEAHQASLPAST
NCU09803.1	----------------------MST-------------IK~DITSIEAWNNHVSSLPAST
FG01317.1 	------------------MPT-----------------IT~EITSITEWEQLLGSVPPTT
AN7567.1  	------MST-----------------------------LF~EVTSEESFGPHLASIPSDT
          	       ::                                ::*:   :    .*:*. *

MG06356.1 	LQIIYFHAPWAPPCAQMATVLKTLSSEYPVTDPLATSWVSLDAEDLSDVSETYDVTAVPF
NCU09803.1	LLIVSFHAPWAAPCAQMATVLKTLASEYPVTEPLSTSWVSIDAEELSEISETYNVTAVPF
FG01317.1 	LVIVSFHAPWAAPCAQMATVLSTLASEYPDTEPPTTKWVSINAEELSDLSETYDVTAVPF
AN7567.1  	LIVLYFHAPWAAPCAQMRAVLSALASQYPATTPPTVSFISVNAEELPDISEEYDVTAVPY
          	* :: ******.***** :**.:*:*:** * * :..::*::**:*.::** *:*****:

MG06356.1 	VVLSRGGKVLESISGNSAQRVRTAIETHTSSSGP-GASSAGAP-----------------
NCU09803.1	LVLARNNQVLETVSGSSAVKVRNAIETHAKKSAQFGTEASEKT-----------------
FG01317.1 	LVLLRNGQVVETVSGSSAVKVRTAIETQAKQSGE-NAAASGPN-----------------
AN7567.1  	VVLLRNGQVLETISGSEATKVRDAVERYAG-AGSAGASANGAASAIPPALTAVPREDVNT
          	:** *..:*:*::**..* :** *:*  :  :.  .: :    :: ..: :: . .. .:

MG06356.1 	--VAASDEAAKAEG-----APQDPEKQKEELFKRLADLVKAAPVMLFMKGTPSAPQCGFS
NCU09803.1	--PAIANGAATAEGQAGDVVPQDPKKQKEELFRRLGDLVKAAPVMLFMKGTPSEPKCGFS
FG01317.1 	--GVAADDAVVEEE-------QDPEKKKEELFKRLGDLVKAAPVMLFMKGTPSSPQCGFS
AN7567.1  	PTTATQAPVAGAASGAGAAPALTPEQSKEALFARLSELVKAAPVMLFMKGTPSAPQCGFS
          	.: .    ..     :.   .  *::.** ** **.:**************** *:****

MG06356.1 	RQLVALLRENSVKYGFFNILADDEVRQGLKEFADWPTYPQLWMDGELVGGLDI0VKEEIG
NCU09803.1	RQLVAILRENAVKYGFFNILADDEVRQGLKEFADWPTYPQLWVDGELVGGLDI0VKEELA
FG01317.1 	RQLVGLLRDNSVKYGFFNILADDEVRQGLKEFADWPTYPQLWIDGELVGGLDI0VKEEMS
AN7567.1  	RQLVGILRERSVKYGFFNILADEDVRQGLKEFADWPTFPQLWVNGELVGGLDI0VKEELE
          	****.:**:.:***********::*************:****::********* ****: 

MG06356.1 	NDPDFFKAYSVKASTDA-AGTS
NCU09803.1	NDADFFKPYSVKANGDASAGQS
FG01317.1 	NDAEFLTKYSVSAPAAP-----
AN7567.1  	NDPNFLDSFSINK---------
          	**.:*:  :*:.
```
